# Analysis of Compositional Bias in a Commercial Phage Display Peptide Library by Next-Generation Sequencing

**DOI:** 10.3390/v14112402

**Published:** 2022-10-29

**Authors:** Ane Beth Sloth, Babak Bakhshinejad, Malte Jensen, Camilla Stavnsbjerg, Mikkel Baldtzer Liisberg, Maria Rossing, Andreas Kjaer

**Affiliations:** 1 Department of Clinical Physiology and Nuclear Medicine & Cluster for Molecular Imaging, Copenhagen University Hospital—Rigshospitalet & Department of Biomedical Sciences, University of Copenhagen, 2200 Copenhagen, Denmark; 2 Nano-Science Center, Department of Chemistry, University of Copenhagen, Universitetsparken 5, 2100 Copenhagen, Denmark; 3 Center for Genomic Medicine, Rigshospitalet, Copenhagen University Hospital, 2100 Copenhagen, Denmark; 4 Department of Clinical Medicine, Faculty of Health and Medical Sciences, University of Copenhagen, 2200 Copenhagen, Denmark

**Keywords:** biopanning, compositional bias, deep sequencing, departure from randomness, M13 phage, next-generation sequencing, phage display, Ph.D.^TM^-12 peptide library, principal component analysis

## Abstract

The principal presumption of phage display biopanning is that the naïve library contains an unbiased repertoire of peptides, and thus, the enriched variants derive from the affinity selection of an entirely random peptide pool. In the current study, we utilized deep sequencing to characterize the widely used Ph.DTM-12 phage display peptide library (New England Biolabs). The next-generation sequencing (NGS) data indicated the presence of stop codons and a high abundance of wild-type clones in the naïve library, which collectively result in a reduced effective size of the library. The analysis of the DNA sequence logo and global and position-specific frequency of amino acids demonstrated significant bias in the nucleotide and amino acid composition of the library inserts. Principal component analysis (PCA) uncovered the existence of four distinct clusters in the naïve library and the investigation of peptide frequency distribution revealed a broad range of unequal abundances for peptides. Taken together, our data provide strong evidence for the notion that the naïve library represents substantial departures from randomness at the nucleotide, amino acid, and peptide levels, though not undergoing any selective pressure for target binding. This non-uniform sequence representation arises from both the M13 phage biology and technical errors of the library construction. Our findings highlight the paramount importance of the qualitative assessment of the naïve phage display libraries prior to biopanning.

## 1. Introduction

During recent decades, the merge of combinatorial chemistry and biological selection has led to the birth of biological combinatorial libraries, which have revolutionized the methodology of ligand discovery [1]. These libraries have facilitated the screening of large pools of randomly generated molecules and allowed for the selection of specific ligands from a huge variety of genetic variants in one test tube. Phage display, first introduced in 1985 by George Smith [2], is recognized as the most commonly used combinatorial library strategy for the identification of novel peptides toward biological targets. In this approach, the in-frame fusion of exogenous oligonucleotides into one of the phage surface protein genes results in the expression of foreign peptides as part of the relevant coat protein. Thus, a physical association is created between the displayed peptide (phenotype) and the corresponding genetic information in the phage genome (genotype), which forms the basis of phage display library selection. The surface display enables peptides to interact with target molecules, giving rise to the selection of clones whose displayed peptides bind specifically to the target [3]. Commercial libraries have played a key role in expanding the scope of the phage display field and a vast number of peptide discovery studies have been conducted based on these libraries. Both filamentous and lytic phages have been used to develop commercial phage display libraries provided by New England Biolabs (M13), Creative Biolabs (M13, T4, and T7), and Novagen (T7). The Ph.D.^TM^ libraries, developed by New England Biolabs (NEB), are the most widely used commercial phage display peptide libraries.

Library construction is an important step in the phage display workflow. The probability of finding variants with desired properties is significantly dependent on the quality of the library, which is mainly defined by the library diversity [4,5]. A fundamental prerequisite for a high-quality and high-diversity library is the random representation of amino acids in the constructed naïve library [6]. The random distribution of amino acids ensures that the identified peptides have become enriched in the recovered pool due to a genuine selection, and not in a biased target-independent manner. Divergence from this randomness can lead to overrepresentation, underrepresentation, and censorship of some peptide sequences in the naïve library. Overrepresentation can result in the propagation-related enrichment of nonspecific peptides during panning, whereas underrepresentation and censorship can deplete the library of some promising hits. Another type of compositional bias that might exist in the constructed library is the high number of clones without inserts, i.e., wild-type clones. These clones do not display any peptides and are regarded as an artifact of library construction.

It is of paramount importance to assess the quality of the constructed library prior to biopanning. This is done through DNA sequencing of the library clones. Traditionally, Sanger sequencing has been used to analyze the library quality [7]. The major shortcoming of this approach is that only a tiny fraction of the library (up to a few hundred clones) is subjected to sequence analysis. The emergence of high-throughput sequencing, also known as next-generation sequencing (NGS), technologies have substantially contributed to the area of phage display research. NGS improves the analysis of phage display libraries by sequencing thousands to millions of clones simultaneously which provides a more detailed understanding of the library quality [8]. In the current study, we used NGS to characterize the commercial Ph.D.^TM^-12 library offered by NEB. This library displays linear 12-mer peptides and has been used extensively for finding target-specific hits [9,10,11]. Our NGS-based analysis indicated that the naïve library contains compositional bias, reflected by the non-random representation of sequences at DNA, amino acid, and peptide levels as well as a high frequency of wild-type clones. Based on these results, we discuss how these deviations from randomness are significant for library screening and can negatively impact the selection of genuine binders during cyclic rounds of bio-panning.

## 2. Materials and Methods

### 2.1. Phage Display Peptide Library

Ph.D.^TM^-12 phage display peptide library (Lot number.: 10111202) was purchased from New England Biolabs (Ipswich, MA, USA). The library has been constructed based on the M13KE phage vector using NNK mutagenesis.

The M13KE phage vector is a derivative of the M13mp19 vector, which allows for the construction and rapid propagation of phage display libraries using standard M13 techniques. The M13KE phage displays a diverse pool of random 12-mer peptides in a pentavalent manner as N-terminal fusions to the minor coat protein pIII of the filamentous M13 phage. The first amino acid of peptide-pIII is the first randomized position of the displayed ligand. The library contains a short linker sequence (Gly-Gly-Gly-Ser) between the displayed peptide and the mature pIII, which improves the target accessibility to the displayed peptide. The reported complexity of Ph.D.^TM^-12 library is on the order of 10^9^ individual clones.

### 2.2. Titering of Phage Suspensions

An aliquot of one or two µL of phage library was used to prepare serial dilutions in liquid LB medium. The phage dilutions were added to the mid log-phase culture of the *Escherichia coli* strain ER2738 (a robust F+ bacterium) from New England Biolabs (Ipswich, Massachusetts, USA). After 5 min, 3 mL of top agar (45 °C) was added to the infected bacterial cells and the suspension of phage-bacterium was poured onto pre-warmed LB/IPTG/Xgal plates (IPTG: isopropyl-β-D-1-thiogalactopyranoside; Xgal: 5-bromo-4-chloro-3-indolyl-β-D-galactopyranoside both purchased from Thermo Fisher Scientific, Waltham, MA, USA). The plates were kept overnight at 37 °C and blue plaques were counted the next day. The number of blue plaques was used to calculate the titer of the library and the mean of two titrations was used.

### 2.3. Illumina High-Throughput Sequencing

Single-stranded DNA (ssDNA) was isolated from the Ph.D.^TM^-12 library using NucleoSpin^®^ Plasmid, Mini kit for plasmid DNA (Macherey-Nagel, Düren, Germany) omitting the amplification step in *E. coli* in the manufacturer’s protocol. A PCR reaction was performed with 100 ng of DNA, Q5^®^ High-Fidelity 2X Master Mix (New England Biolabs, Ipswich, MA, USA), and forward and reverse primers (0.5 μM). The primers consist of an overhang containing the adapter and a target-binding region (underlined).

Forward primer: 5′-TCG TCG GCA GCG TCA GAT GTG TAT AAG AGA CAG ACC TCG AAA GCA AGC TGA TAA ACC G-3′

Reverse primer: 5′-GTC TCG TGG GCT CGG AGA TGT GTA TAA GAG ACA GCT GTA GCA TTC CAC AGA CAG CCC-3′

PCR cycling conditions were 98 °C for 30 s, followed by 5 cycles of 98 °C for 10 s, 60 °C for 30 s, 72 °C for 20 s, and a final extension at 72 °C for 2 min. Amplified DNA was purified using the GeneJET PCR Purification Kit (Thermo Fisher Scientific, Waltham, MA, USA) according to the manufacturer’s instructions. For the indexing PCR, 25 ng of purified PCR product, 5 μL of i7 and 5 μL of i5 Nextera XT indexing primers (Illumina, San Diego, CA, USA), and 25 μL of Q5^®^ High-Fidelity 2X Master Mix were run in a total volume of 50 µL. Cycling conditions were initial denaturation at 95 °C for 3 min and 8 cycles of 98 °C for 30 s, 60 °C for 30 s, 72 °C for 30 s, and a final extension at 72 °C for 5 min. The indexed PCR product was purified using the GeneJET PCR Purification kit. After each PCR, the product was analyzed using BioAnalyzer 2100 DNA 1000 Kit (Agilent, Santa Clara, CA, USA) to verify the quantity and quality.

The sample underwent quality control using the Fluoroskan^TM^ Microplate Fluorometer (Thermo Fisher Scientific, Waltham, MA, USA) with the Quant-iT^TM^ 1X dsDNA HS Assay Kit (Thermo Fisher Scientific, Waltham, MA, USA). The indexed sample was sequenced using NextSeq 500/550 Mid Output Kit v. 2.5 (Illumina, San Diego, CA, USA) using 250-bp single-end sequencing. The individual base call (BCL) file was demultiplexed and the FASTQ file was generated using the bcl2fastq software, provided by Illumina. Illumina sequencing and the generation of the FASTQ file were conducted by the Center for Genomic Medicine, Rigshospitalet, Copenhagen University Hospital, Denmark.

### 2.4. Analysis of High-Throughput Sequencing Data

A MATLAB script was used to process the resulting FASTQ file as previously described in [12] (with some modifications). The script used for data processing is available on GitHub (https://github.com/KjaerLab/Compositional-bias-in-a-commercial-peptide-phage-display-library-git, accessed on 29 September 2022). The analysis of the sample was performed by defining the borders of the variable region in the Ph.D.^TM^-12 library. Afterward, this region was converted into amino acids, and a frequency-sorted matrix was made containing each amino acid sequence, its occurrence, and its frequency. The reads containing ‘*’ or ‘X’ or not containing the GGGS-linker were all removed and put into a frequency sorted matrix of their own (removed reads). The final data consist of a frequency-sorted matrix containing the clean reads with their amino acid sequence, their occurrence, and their frequency. Raw data can be found at https://sid.erda.dk/share_redirect/BeSx4GLjb4 (accessed on 29 September 2022).

The resulting comma-separated values (.csv) files were used for subsequent analysis by an in-house Python script in Jupyter Notebook, available on GitHub (https://github.com/KjaerLab/Compositional-bias-in-a-commercial-peptide-phage-display-library-git, accessed on 29 September 2022). The amino acids were encoded by one hot encoding for numerical analyses. The global frequency of each amino acid was calculated using the Python script. Principal Component Analysis (PCA) was performed in Python for all unique sequences. The clustering was performed on the projection onto the first two principal components using the Gaussian Mixture Model. The PCA finds the co-occurrence of multiple positional patterns at once and is multi-dimensional. Heatmaps were generated for each individual cluster as well as for the whole dataset. They show the positional frequency and were created using the Python script.

## 3. Results

### 3.1. There Is a Discrepancy between in-House and NEB Titers of the Ph.D.^TM^-12 Library

Before performing the experiments, the Ph.D.^TM^-12 Phage Display Library from NEB was titered using the plaque-count method. This titering revealed a discrepancy to the titer reported by NEB (2.3 × 10^11^ pfu/mL compared to the reported 1 × 10^13^ pfu/mL). NEB states that the Ph.D.^TM^-12 library consists of around 10^9^ unique sequences. Assuming equal copy numbers for all variants of the library, each unique sequence has 100 copies in 10 µL (10^11^ pfu), which is the standard recommended input for biopanning. However, the in-house titer determination suggests that there are only 2.3 copies of each unique sequence in 10 µL.

### 3.2. Variable Region Contains Some Deviations from NNK Randomization

**Figure 1:**
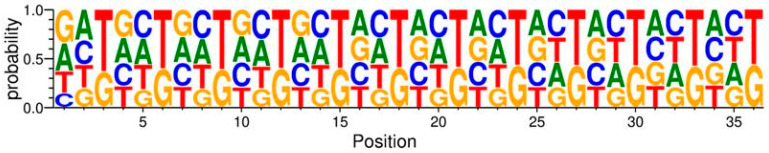
Sequence logo showing the 36 nucleotides encoding the 12-mer variable region with a probability of 0–1. The height of each nucleotide represents the probability of its occurrence in the given position. The sequence logo was generated using the WebLogo 3 library in Python.

To investigate potential biases at the level of nucleotides, the codons encoding amino acids in the variable region were visualized with a sequence logo (Figure 1), where the probability of each nucleotide is represented by the height of the letters. This was conducted by the WebLogo 3 library in Python [13,14]. The Ph.D.^TM^-12 library is generated using the NNK strategy, where N denotes all four nucleotides (A, T, C, G) and K represents G or T. The NNK strategy improves the representation of every canonical amino acid, and the number of codons is reduced to 32 [15]. The advantage of this randomization scheme is that only one stop codon exists in the library. In this approach, cysteine, aspartic acid, glutamic acid, phenylalanine, histidine, isoleucine, lysine, methionine, asparagine, tyrosine, and tryptophan are encoded by a single codon, while glycine, alanine, glutamine, leucine, proline, arginine, serine, threonine, and valine are encoded by two or three codons [16]. In the Ph.D.^TM^-12 library, the remaining stop codon, TAG, is suppressed by the host bacterial strain. Therefore, glutamine is encoded by two codons: CAG as its natural codon and the stop codon TAG.

As seen in Figure 1, the variable region follows the NNK structure with all codons having either G or T in the third position. Furthermore, all four nucleotides are present in the first and second positions of the codons. However, there are some deviations from NNK randomization. Generally, T has a higher probability compared to G in the third position of each codon. G has a lower probability than other nucleotides at the second position of many codons. At position one of the first codon, the presence of G and A has a higher probability compared to T and C.

### 3.3. Filtering of Sequencing Data Results in the Removal of More Than 20% of Reads

The raw FASTQ file was analyzed by an in-house MATLAB^®^ script, removing reads that do not contain the GGGS linker, do not encode a 12-mer peptide, and all sequences containing ‘*’ and ‘X’. There was a total of 6,940,916 reads, whereas 5,418,602 reads were kept, and 1,522,314 reads (21.9%) were removed. The Python script was used to analyze the pool of removed reads. In the removed pool, 5233 reads contained frameshifts. A frameshift was defined as a shift of one or two nucleotides either upstream or downstream of each end of the variable region, which did not result in a variable region of 48 nucleotides or an insert divisible by three. Multiple inserts of the variable region were defined as sequences with insertion of the entire 48 nucleotides of the variable region multiple times. This can be observed once in our dataset. An additional sequence of 55 nucleotides has been inserted 204 times. However, according to our definition, it does not qualify as a clone with multiple inserts. Reads containing the stop codon TAG make up 8% of all removed reads. In our analysis, TAG is not translated into glutamine, and therefore these reads result in a ‘*’, causing them to be removed. The vast majority of removed reads originated from wild-type clones, which are clones without the insert. These variants comprise 44% of the removed reads (670,000 reads).

### 3.4. The M13KE Wild-Type Clone Is Highly Abundant in the Ph.D.^TM^-12 Library

**Figure 2:**
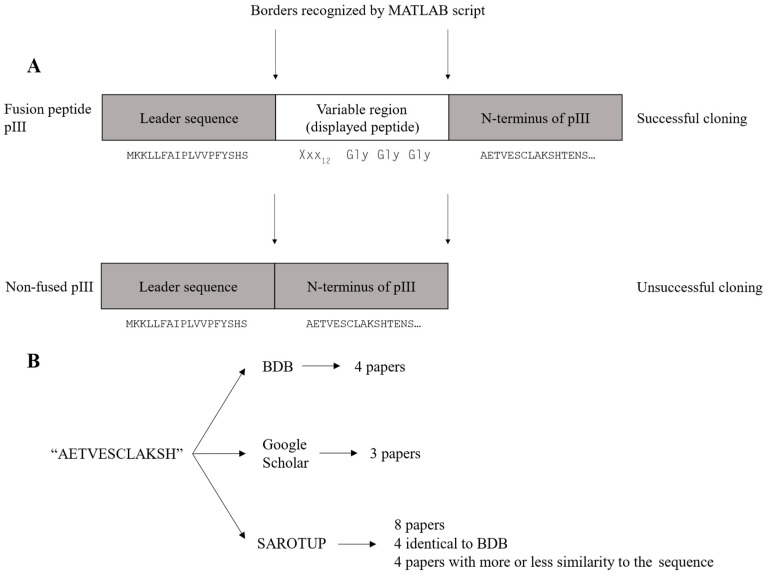
(**A**) An overview of the fusion peptide pIII in the case of successful and unsuccessful cloning of the variable region into the M13KE vector. MATLAB script recognizes a fragment corresponding to the borders of the variable region, displaying the peptide. In the case of unsuccessful cloning of the variable region, the area recognized by the script will correspond to the N-terminus of pIII, resulting in reads with the sequence: AETVESCLAKSHTENS. (**B**) Flow chart of literature search for the ‘AETVESCLAKSH’ sequence.

The wild-type clone constitutes a major fraction of the removed reads as mentioned above. The wild-type clone is observed in the dataset as the sequence AETVESCLAKSH. This sequence is identical to the N-terminus of the mature pIII, located immediately downstream of the displayed peptide in the M13KE cloning vector (Figure 2A). The wild-type clone arises due to the unsuccessful cloning of the variable region into the M13KE vector during library construction. The titer reported by NEB is the sum of both peptide-displaying and wild-type clones. Given the high abundance of the wild-type clone in the library, both the library diversity and the copy number of each variant are reduced further.

As NGS analysis allows us to exclude the AETVESCLAKSH sequence as an artifact of the phage display library construction, a literature search was conducted to investigate if the AETVESCLAKSH sequence had been reported as a target-binding peptide in the literature. The literature search was performed using Biopanning Data Bank (BDB) [17], Google Scholar, and SAROTUP (using the MimoBlast tool) [18]. The literature search flow chart is shown in Figure 2B, and the results are summarized in Supplementary Table S1. AETVESCLAKSH (or AETVESC in the case of Ph.D.^TM^-7) was found in all three combinatorial libraries from NEB (Ph.D.^TM^-12, Ph.D.^TM^-C7C, and Ph.D.^TM^-7), and the sequence has been reported to show target binding in some of the papers [19,20]. Statistically speaking, the probability of the presence of the AETVESCLAKSH peptide within the Ph.D.^TM^ phage display libraries is quite low. Additionally, the isolation of this peptide in biopanning on different targets casts doubt over the genuine target binding of this sequence. It seems that this peptide has not been distinguished from the N-terminus of mature pIII, leading to the misidentification of AETVESCLAKSH as a target-binding peptide.

### 3.5. Global Frequencies of Amino Acids Differ from the Expected Frequencies

**Figure 3:**
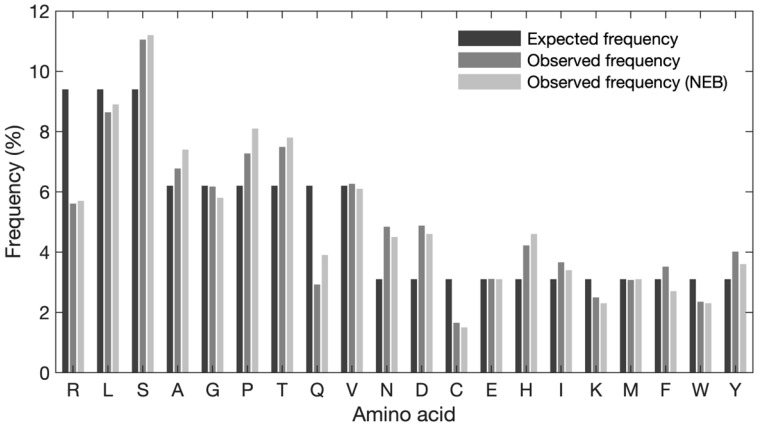
Global frequency of each amino acid (one-letter code). The expected frequency calculated based on available codons is compared to the observed frequency from our dataset and to the observed frequency provided by NEB. The frequency of glutamine (Q) does not include those encoded by the TAG codon. The frequency of unaccounted Q is 0.4 percentage points.

To characterize and investigate potential sequence bias in the naïve library, an analysis of the amino acid composition was performed. First, the global frequency of each amino acid was evaluated (Figure 3). The expected frequency of each amino acid was calculated based on the number of available codons, when obeying the NNK structure (e.g., 3 codons/32 codons × 100% = 9.4%, 2 codons/32 codons × 100% = 6.3%, 1 codon/32 codons × 100% = 3.1%). Our data show some discrepancies between the expected and observed frequencies of amino acids (see Supplementary Table S2 for exact frequencies). Serine, proline, threonine, asparagine, aspartic acid, and histidine are observed at higher frequencies than expected and arginine, glutamine, and cysteine are observed at lower frequencies than expected. When comparing the observed frequency with the expected frequency, asparagine and aspartic acid exhibit the largest degree of overrepresentation (approx. 1.75% increase for both), whereas arginine shows the largest degree of underrepresentation (3.8% decrease). Overall, serine is the most frequently observed amino acid (11.04%) and cysteine is the least frequent residue (1.65%) Of note, the observed frequencies reported by NEB and our in-house data exhibit a similar pattern; however, for alanine, proline, and glutamine differences can be observed.

### 3.6. Positional Frequencies of Amino Acids Reveal Major Biases from the Expected Frequencies

**Figure 4:**
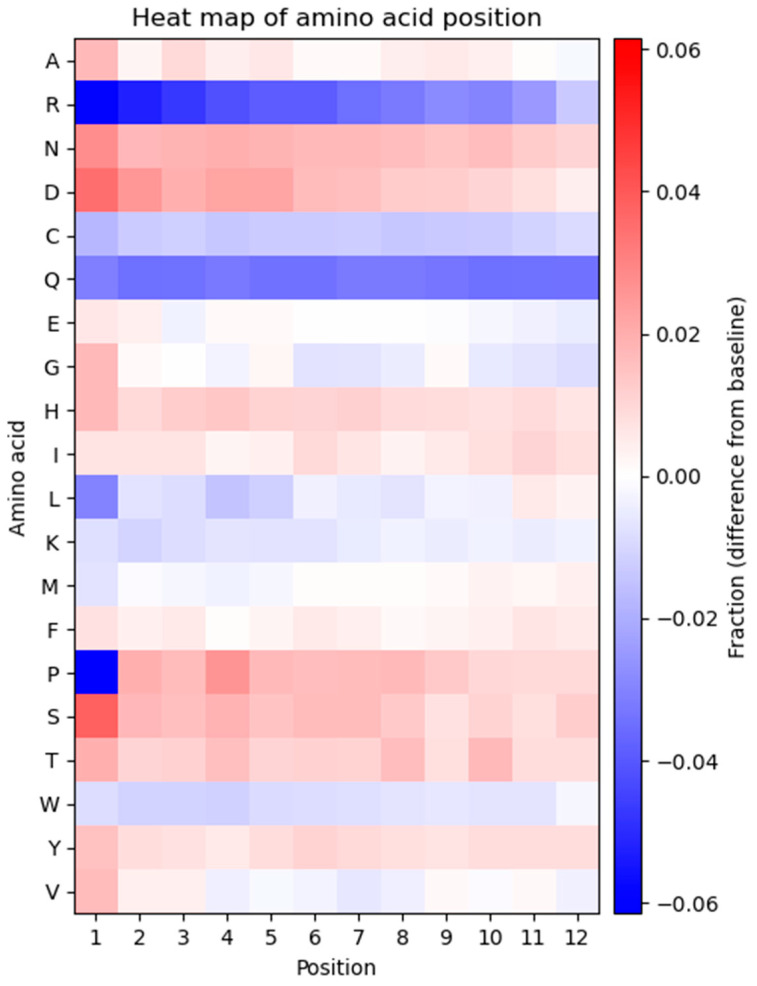
Heatmap showing the positional frequency of every amino acid in each position throughout the displayed peptide compared to the expected frequency, calculated based on the codons available. Overrepresented amino acids (red) and underrepresented amino acids (blue) according to the expected frequency are shown in the heatmap.

The frequency of each amino acid in every position of the displayed peptide sequence was determined to investigate potential bias in the positional frequencies (Figure 4). The heatmap represents the observed frequency of every amino acid in each position and is compared to the expected frequency, calculated based on the number of available codons for each amino acid.

As displayed in the heatmap, the positional frequency of some amino acids is not consistent across all residues, and several positional biases can be observed. A general trend of the heatmap is that every amino acid displays one type of bias, either overrepresentation or underrepresentation in every position. The only exceptions are glycine, proline, and valine which show a combination of over- and underrepresented amino acids at different positions. Arginine and glutamine exhibit underrepresentation compared to the expected frequency and exhibit considerable bias in most positions. Proline is strongly underrepresented at position one. Aspartic acid and serine are overrepresented in multiple positions. Several residues (alanine, asparagine, glycine, histidine, leucine, lysine, methionine, threonine, tryptophan, and tyrosine) show variations across all 12 positions in the peptide. Glutamic acid, isoleucine, and phenylalanine exhibit a fixed pattern of amino acid positional distribution across all residues.

Another intriguing finding is that the amino acid residues at the N-terminus of the displayed peptide exhibit a greater extent of bias. This can be seen in position 1, which is the most biased position in the displayed peptides. Proline and arginine at position 1 are the most underrepresented and serine at position 1 is the most overrepresented amino acid within the displayed peptide.

### 3.7. Principal Component Analysis Depicts the Presence of Different Subgroups in the Naïve Library

**Figure 5:**
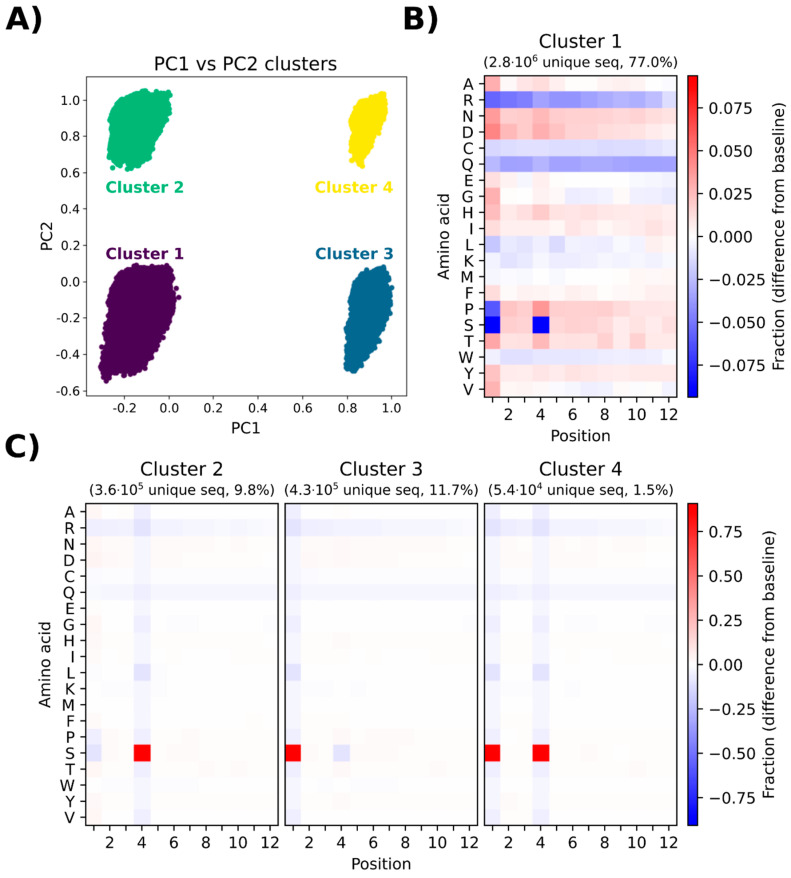
(**A**) PCA plot (PC1 and PC2) of the naïve library showing four clusters in the pool of sequences. (**B**) Heatmap showing overrepresentation (red) or underrepresentation (blue) compared to the expected value of each amino acid for each position according to the expected value for cluster 1. (**C**) Heatmap showing overrepresentation (red) or underrepresentation (blue) compared to the expected value of each amino acid for each position according to the expected value for cluster 2, cluster 3, and cluster 4.

Principal component analysis (PCA) was performed to investigate any potential sequence clustering within the naïve library. The variance is defined as the variation in the dataset, which can be attributed to each principal component (PC). PC1 and PC2 describe the most variance and the second most variance in the dataset, respectively. These two components were therefore used for the analysis, and for the clearest visual representation of the data, a plot was made with just PC1 and PC2. When plotting PC1 towards PC2, four distinct clusters (cluster 1–cluster 4) are displayed. The sequences making up each cluster (Figure 5A) were presented in heatmaps displaying which amino acids are over- or underrepresented in that cluster (Figure 5B,C).

Cluster 1 constitutes the largest portion of the sequenced pool (77%), which is reflected in the heatmap (Figure 5B), showing a complex pool of sequences with considerable position-specific biases for amino acids similar to the general heatmap shown in Figure 4. The major difference is the severe underrepresentation of serine in positions 1 and 4. The other clusters (cluster 2, cluster 3, and cluster 4, Figure 5C) show a relatively uniform position-specific distribution of all amino acids within the entire length of the displayed peptide. The major positional biases include overrepresentation of serine at position 4 for cluster 2, overrepresentation of serine at position 1 for cluster 3, and overrepresentation of serine at positions 1 and 4 for cluster 4. Due to the high degree of similarity between cluster 1 and the naïve library, cluster 1 can be regarded as a good representative of the entire library. A PCA on simulated data was also generated to compare the naïve library with a truly random library, i.e., a dataset generated by simulating a random NNK library (Supplementary Figure S1). In the simulated PCA plot, only one uniform cluster is observed and the heatmap indicates insignificant position-specific differences for each amino acid. The results of PCA provide evidence for a non-random representation of amino acids in the library, which leads to the emergence of different subpopulations in the library without the presence of any selective pressure associated with biopanning.

### 3.8. The Naïve Library Is Not Uniform and Contains Peptides at High Frequencies

In a uniform and random library, all peptide sequences would have an equal abundance. NEB states that each library consists of 10^9^ unique clones. Using an input of 10^11^ pfu (according to NEB titer) and assuming an equal number of each clone, this results in 100 copies of each clone. However, our NGS analysis revealed the presence of peptides with a broad range of frequencies and some peptides had significantly higher copy numbers than expected.

**Figure 6:**
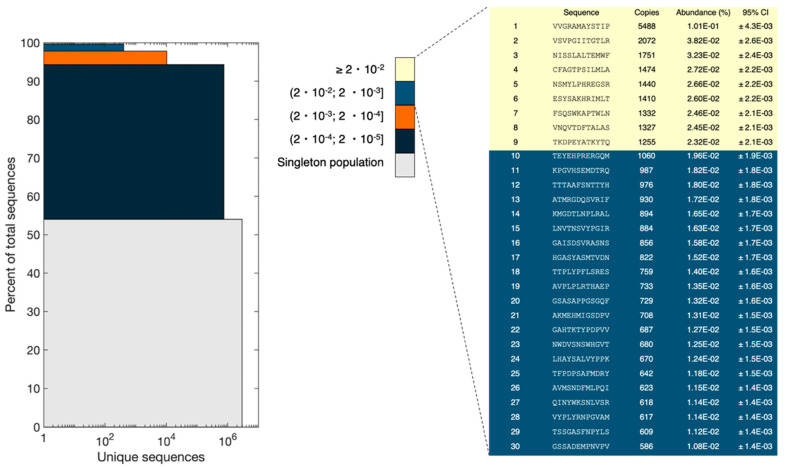
Stacked bar plot with color-coded segments grouping sequences according to their abundance (see color key). The height of each segment illustrates the sum of abundance for each segment and the width corresponds to the number of unique sequences in each segment. Grey: 2,927,839 reads, 54%; dark blue: 752,541 reads, 40.3%; orange: 10,258 reads, 3.5%; light blue: 406 reads, 1.9%; yellow: 9 reads, 0.3%. The top 30 peptides are shown on the right and colored according to the bin they are included in. The 95% confidence intervals with Bonferroni correction are shown to evaluate the validity of the ranking.

The resulting reads obtained from NGS were divided into bins of sequence abundance intervals and the data were represented in a stacked bar plot (Figure 6), as done in Matochko et al. [21]. For the sequences only observed once (singleton population), the sum of abundances was 54% of the total pool (corresponding to 2,927,839 reads), which is the biggest population in the pool of sequences. The sum of abundances of the most frequent sequences (frequency ≥ 0.02%) comprised 0.3% of the population. However, the most frequent peptide had a frequency of 0.1% and 5488 copies. Several peptides had more than 500 copies. Assuming a binomial distribution of the subsampling of the phage pool, the probability of observing one clone once in our experimental setup would be 0.0054 (5,418,602 reads and 10^9^ different peptides). The probability of observing two or three copies is 1.46 × 10^−5^ and 2.64 × 10^−8^, respectively. This means that approximately 0.54% of the library is represented with a single copy, 0.00146% of the library is represented with two copies, and only 2.64 ×10^−6^% of the library has three copies of the same peptide. Therefore, observing more than 500 copies for several peptides is statistically unlikely and is the result of bias in the naïve library. It is not possible to observe the full diversity of the library, as the number of possible reads is lower than the number of variants present in the library. It is also worth mentioning that sampling bias associated with NGS sample preparation and sequencing might result in some changes in the peptide pool composition.

To estimate the validity of the ranking shown in Figure 6, the 95% confidence interval for peptides in the top 30 was calculated with Bonferroni correction. Confidence intervals were calculated using a normal approximation. The standard deviation is calculated as σ = √(np(1 − p)), where n is the number of reads from the NGS, and p is the observed probability of each unique sequence. When the criteria, p × n and (1 − p) × n is equal to or greater than 5, are met, the normal distribution will be a sufficiently good approximation for the binomial distribution [22]. For all experiments, the criteria were met. Bonferroni correction, which is a conservative approach, was used to adjust for multiple testing of the confidence intervals. Based on these data, the peptides in ranks 1, 2, and 3 are statistically certain. However, peptides in ranks 4–9 have overlapping confidence intervals, and their rankings are not statistically certain. Thus, they could be interchangeable. Additionally, peptides in ranks 10–30 have overlapping confidence intervals. Therefore, we cannot conclude that their rank in Figure 6 would be conserved in a similar experiment. However, we are certain that the peptides with ranks 10–30 do not belong to the peptides in ranks 4–9 as well as ranks 1–3.

## 4. Discussion

The principal presumption of phage display biopanning is that the naïve library contains an unbiased repertoire of peptides, and thus, the enriched variants derive from the affinity selection of a fully random peptide pool [6]. To the best of our knowledge, this work is the first in-depth characterization of the commercial Ph.D.^TM^-12 phage display library using high-throughput sequencing. We first titered the library and then conducted a series of analyses on the library composition. The NGS analysis of the library composition demonstrated a remarkable sequence bias at DNA, amino acid, and peptide levels. Our in-house titration of the naïve library by plaque count method showed a 43-fold lower number of phage virions compared to NEB-reported titer. Given that the library consists of a non-uniform distribution of peptides with a wide variation in copy numbers (Figure 6), the observed reduced titer might lead to the exclusion of some promising variants with low copy numbers. We had already detected a decreased titer for the Ph.D.^TM^-7 phage display library from NEB, suggesting it to be a general problem for different libraries (data not published). Due to the magnitude of titer reduction and the fact that it has been observed in different libraries, it cannot be attributed to storage conditions. We highly recommend that the users of these libraries determine the titer in their labs and use the in-house titer as a basis for experiments.

The Ph.D.^TM^-12 library was constructed using NNK codon randomization. The utilization of this method eliminates two stop codons (ochre and opal) and the library variants are encoded with only 32 codons, which can insert all 20 amino acids into the displayed peptides [16]. The remaining amber stop codon (TAG) is suppressed by propagating the phage in a *supE E. coli* strain that incorporates glutamine into the position of the amber codon during translation of phage proteins [23]. A high-quality library should contain a low number of variants with stop codons, frameshifts, multiple inserts, as well as wild-type variants. Our data showed that 8% of NGS reads contained amber stop codons, while there were no ochre and opal stop codons as expected. The detection of stop codons might either result from sequencing errors [24] or derive from DNA debris existing in the extracted phage pool that is associated with the lack of complete efficiency of amber suppression by the host bacterium [25]. Therefore, as a conservative measure to reduce the risk of false-positive peptide leads, TAG is not substituted with glutamine in our data analysis, thus causing an underrepresentation of glutamine compared to the expected frequency (Figure 3). Additionally, 0.075% of reads contained frameshift mutations, likely introduced during the chemical synthesis of randomized oligos. We did not find any clones with multiple inserts. The presence of stop codons and frameshifts in the variable region leads to no production of infectious phage virions [15]. Furthermore, our deep sequencing analysis revealed a high abundance of wild-type clones in the Ph.D.^TM^-12 library. This is caused by inefficient linearization of the M13KE vector molecules by restriction endonucleases, which might be associated with low stringency of vector digestion as well as the presence of variable forms of vector DNA extracted from infected bacterial cells [8]. This problem can be overcome by enhancing the stringency of the digestion reaction or by using methods, such as sucrose gradient density centrifugation, for the purification of the digested vector [26]. As pIII plays a critical role in M13 phage infection and assembly [27], the insertion of randomized peptides into this protein can impose a burden on phage propagation. The lack of insert removes the probable structural constraint, caused by many displayed peptides, on the membrane translocation of the pIII fusion protein and its subsequent folding in the periplasmic space and, thus, wild-type clones gain a propagation advantage compared to the pool of peptide-displaying viruses. The acquired propagation advantage results in the enrichment of wild-type clones during biopanning in a target-independent manner. The corruption of the library by the strong enrichment of the wild-type clone hampers several rounds of selection because the wild-type clone out-competes the target-related clones. This is a bigger problem when using Sanger sequencing since the enrichment of wild-type clones, particularly in earlier rounds, can easily escape detection by low-throughput sequencing. Altogether, the existence of stop codons, frameshifts, and wild-type clones affects the effective size of the library. Assuming the library has 1 × 10^9^ independent transformants and contains 9.65% wild-type clones, 8% oligonucleotides with stop codons, and 0.075% frameshift mutations, the effective size of the library approximately equals 8.23 × 10^8^ different peptides, according to similar calculations by Kulseth et al. [7].

The investigation of the nucleotide distribution of the library indicated compliance with NNK randomization. However, there are some deviations from randomness in the first, second, and third positions of each codon (Figure 1). This can lead to some biases at the amino acid level, such as favoring amino acids encoded by codons with T at position three (F, I, Y, H, N. D, and C). Apart from cysteine, this is reflected in the global frequency of amino acids (Figure 3). These imbalances in the distribution of nucleotides can influence the amino acid composition of displayed peptides. A minor fraction of these distortions from true randomness might result from biased chemistries in the oligonucleotide synthesis step, as already shown in the production of aptamer libraries [28].

The manufacturer has already noticed considerable differences between the theoretical and expected amino acid composition of the naïve library after deep sequencing with Ion Torrent^TM^ technology [29]. Our observations were in concordance with the manufacturer’s data with some minor discrepancies (such as lower overrepresentation of A and P as well as higher underrepresentation of Q). This concordance also holds true for S which has the highest frequency and C which has the lowest frequency in both our and NEB data (Figure 3). Previous studies have reported a sequence bias against unpaired cysteines, leading this amino acid to be censored at all positions throughout the displayed peptide. This censorship is due to the ability of unpaired cysteine residues to form an intramolecular disulfide bond (S-S) with the intrinsic cysteine in pIII, which can interfere with the assembly or infection of the filamentous phage [30]. Based on this, even numbers of cysteine are considered in designing disulfide-constrained phage display peptide libraries. To generate a library with odd numbers of cysteine, phages with disulfide-free pIII might be useful [31]. Characterization of the Ph.D.^TM^-7 library by ’t Hoen et al. has also shown that the naïve library is considerably depleted of C (frequency < 1%) and P > S > L > T have the highest frequencies [32]. We also found these four amino acids with the highest abundances. However, unlike Ph.D.^TM^-7 library, S is the most abundant amino acid in Ph.D.^TM^-12 library, followed by L, T, and P (Figure 3). Bias is not only dependent on the amino acid sequence but is also dependent on the position of amino acids in the displayed peptide. The low-throughput sequencing of positional diversity in Ph.D.^TM^-12 and Ph.D.^TM^-C7C libraries has shown that the majority of sequence bias in M13 combinatorial phage display libraries is clustered within the N-terminus of the displayed peptide, particularly the first three positions [33]. Our high-throughput sequencing results provide stronger support for this notion, indicating that the first position contains the highest degree of amino acid bias (Figure 4). In line with our data, it has been found that the amino acid sequence of signal peptide and amino acids immediately after the signal sequence (N-terminus of mature pIII) have a major impact on the cleavage rate of signal peptidase and play a determining role in the display level of peptide inserts fused to the M13 coat proteins (such as pIII) [34]. Some peptides suppress cleavage and processing of phage coat proteins, while some others increase sensitivity to the cleavage by signal peptidase and improve the processing of phage coat proteins. The dramatic underrepresentation of proline at position 1 is associated with the cleavage activity of signal peptidase since peptides containing proline immediately downstream of the cleaves site act as inhibitors for the enzyme, and thus, suppress the proteolytic processing of pIII [35,36,37]. If the presence of a specific amino acid sequence is required after the signal peptidase cleavage site, it is recommended to predict the position-specific cleavage—by using tools such as SignalP—to avoid the risks of improper or poor cleavage [38]. We also found that R has the largest underrepresentation in the library (Figure 4). The censorship of positively charged sequences through the Sec pathway is a well-identified phenomenon in phage display. Phages that display R-rich sequences have been demonstrated to have a significantly lower production rate and there is a reverse correlation between the number of R residues and phage production [39,40]. This has also been observed in the Ph.D.^TM^-12 and Ph.D.^TM^ C7C phage display libraries by low-throughput sequencing [33]. The censorship of R is more pronounced at the N-terminus of the displayed peptide, which is cleaved by signal peptidase. The censorship of R results from the secY-dependent secretion of pIII. It has been revealed that the presence of positively charged residues near the cleavage site of signal peptidase can hinder the translocation of pIII across the inner membrane and inhibit the secretion of phage particles [39,40]. If the N-terminal R is required, the use of prlA suppressor strains can overcome the secY-dependent secretion problems [41]. The sequence censorship also depends on the location of library cloning in pIII. Libraries expressed at the N-terminus of the N1 domain of pIII have been shown to censor R-rich sequences, while libraries cloned between the N1 and N2 domains of pIII rescue the R-associated bias [42].

The results of principle component analysis (PCA) and peptide frequency distribution investigation (Figure 5 and Figure 6) provided evidence for a substantial heterogeneity and non-random distribution of peptides in the naïve library. PCA indicated the formation of four distinct clusters, mainly distinguished by underrepresentation or overrepresentation of S in positions 1 and 4 within the 12-mer peptide (Figure 5). Furthermore, additional clusters could have been identified by performing projections on other principal components than the first two. However, it is considered beyond the scope of this manuscript to identify and characterize all possible clusters. Furthermore, the distribution of peptide frequencies was also found to be far from uniform with some peptides being present at higher frequencies than expected (Figure 6). Matochko et al., 2014 had a similar finding in the Ph.D.^TM^-7 library [21]. The presence of different sub-populations and huge bias in the non-homogenous distribution of peptide frequencies highlight that although the naïve library has not undergone any selective pressure for target binding, some peptides show enrichment in the pool. This enrichment most probably happens during the propagation step of library construction. The non-uniform representation of peptides in the naïve library can be attributed to both sequence-dependent and -independent biases. The sequence-dependent bias is based on the fact that some phage clones obtain propagation advantage associated with the specific sequence of displayed peptides and sequence-independent bias has been shown to be caused by the enrichment of some phage clones (and their displayed peptides) that obtain propagation advantage due to mutations in the phage genome out of variable region [43]. Therefore, propagation advantage both extrinsic and intrinsic to the peptide can impact the composition of the library, creates a significant bias at the peptide level, and lead to a heterogenous distribution of peptide frequencies that is far from the desired randomness.

## 5. Conclusions

Our findings suggest that the commercial combinatorial Ph.D.^TM^-12 peptide phage display library has decreased diversity due to reduced titer, high frequency of wild-type clones, a significant number of potential fast-propagating clones, and compositional bias in the displayed sequences. Some part of this bias is inherent and arises from the phage-bacterium crosstalk. This crosstalk refers to the interaction between the phage and the host bacterium and includes all stages of the phage life cycle. In this context, phage binding to the pilus of the host bacterium, ribosomal translation of the phage capsid proteins, insertion of the synthesized proteins into the inner membrane of the host cell, signal peptide cleavage of the fusion phage proteins, assembly of the phage virions, and secretion of the phage particles from the infected bacterial cell are steps in which bias might happen. Peptides that are incompatible with the different steps of the phage life cycle are more likely to be censored. In contrast, peptides that improve the evolutionary fitness of phages during viral morphogenesis tend to be retained in the library. However, some part of this bias results from technical errors happening during library construction Therefore, some corrections in the methodology of oligonucleotide synthesis, cloning of library inserts, transformation of library members into the host bacterium, and the use of efficient suppressor strains can reduce such bias in pIII-based phage display systems. The quality control assays performed by NEB are mostly based on Sanger sequencing of 100 plaques from each lot (personal communications with NEB) which is not sufficient to detect underlying problems within the library. Hence, we wish to inform other phage display researchers of the potential pitfalls of the commercial phage display peptide libraries to reduce the risk of identification of false-positive hits. We highly recommend characterization of the naïve library prior to biopanning experiments as different lots of the library might constitute different levels of bias, leading to a distinct set of peptide sequences.

## Data Availability

Not applicable.

## Supplementary Materials

The following supporting information can be downloaded at: https://www.mdpi.com/article/10.3390/v14112402/s1. Figure S1: Simulation of a naïve peptide phage display library; Table S1: Literature search revealing the identification of AETVESCLAKSH in phage display experiments; Table S2: Global frequencies- the expected and observed frequencies by NEB and our in-house analysis.

## Abbreviations

BDB: Biopanning Data Bank, bp: Base pair, IPTG: Isopropyl β- D-1-thiogalactopyranoside, NEB: New England Biolabs, NGS: Next Generation Sequencing, pIII: Filamentous phage protein III, PCA: Principal Component Analysis, PCR: Polymerase Chain Reaction, pfu: Plaque forming unit, Ph.D.^TM^-12: Phage Display Library expressing 12-mer peptide, SAROTUP: Scanner Additionally, Reporter Of Target-Unrelated Peptides, X-gal: 5-bromo-4-chloro-3-indolyl-βeta-D-galactopyranoside.
