# Supplementary material for: Analysis of Compositional Bias in a Commercial Phage Display Peptide Library by Next-Generation Sequencing
Source: Viruses. 2022 Oct 29;14(11):2402. doi: 10.3390/v14112402 (PMC9697088; doi:10.3390/v14112402)
Supplement: Supplementary file 1 [file viruses-14-02402-s001.zip › viruses-1971227-supplementary.pdf]

Supplementary Materials

Table S1: Literature search revealing the identification of AETVESCLAKSH in phage display experiments
Summary of the literature search for the identification of AETVESCLAKSH in phage display experiments. Database, phage display library, peptide sequence, sequencing method, if the peptide has a reported function, which binding assays were performed using the clone/peptide, and the reference for each paper are listed. Differences in peptide sequence compared to the AETVESCLAKSH sequence are marked in bold. Abbreviations: BDB: Biopanning Data Base, ELISA: enzyme-linked immunosorbent assay, FRET: Fluorescence Resonance Energy Transfer, WT: wild-type, SPR: Surface Plasmon Resonance.

Database used	Phage display library	Peptide sequence	Sequencing method	Reported function	Target	Assays	Reference
BDB	Ph.D.-12	AETVESCLAKSH	Sanger	Yes	IgE of melon-allergic patients	ELISA	[20]
BDB	Ph.D.-12	AETVESCLAKSH	Sanger	Yes (part of consensus sequence)	Bacillus cereus 4342	ELISA, Dot-blot assay, Qdot, Coomassie staining	[21]
BDB	Ph.D.-C7C	AETVESCLAKSH	Sanger	No	Type IV secretion system protein VirB8	FRET	[22]
BDB	Ph.D.-12	AETVESCLAKSH	Sanger	No, identified as WT	Gamma-irradiated Salmonella spp.	None	[23]
Google Scholar	Ph.D.-12	AETVESCLAKSH	Sanger	No	TiO_2 nanoparticles	FRET	[24]
Google Scholar	Ph.D.-12	AETVESCLAKSH	Sanger	Yes	CD40 extracellular domain	SPR	[18]
Google Scholar	Ph.D.-12	AETVESCLAKSH	Sanger	No	γδ TCR	None	[25]
MimoBlast	Ph.D.-12	AETVESCLAK**P**H	Sanger	No	PL9-11 panel of IgG anti-DNA antibodies	None	[26]
MimoBlast	Ph.D.-12	**FRSF**ESCLAKSH	Sanger	Yes	Human IL-10 R α protein	ELISA, SPR	[19]
MimoBlast	Ph.D.-7	AETVESC	Sanger	No	IgE from Ara h 1 serum	None	[27]
MimoBlast	Ph.D.-7	AETVESC	Sanger	No	Murine digestive tract	None	[28]

Table S2: **Global frequencies – the expected and observed frequencies by NEB and our in-house analysis**
The global frequency of each amino acid in the naïve Ph.D.^{TM}-12 library is indicated in the table. The expected frequency of each amino acid was calculated by the available number of codons. The observed frequency reported by NEB, as well as the in-house observed frequency is shown.

Amino acid	Code	Expected frequency	Observed frequency (NEB)	Observed freq (in-house)
Arginine	R	9.4	5.7	5.6
Leucine	L	9.4	8.9	8.6
Serine	S	9.4	11.2	11.0
Alanine	A	6.2	7.4	6.8
Glycine	G	6.2	5.8	6.2
Proline	P	6.2	8.1	7.3
Threonine	T	6.2	7.8	7.5
Glutamine	Q	6.2	3.9	2.9
Valine	V	6.2	6.1	6.3
Asparagine	N	3.1	4.5	4.8
Aspartic acid	D	3.1	4.6	4.9

Cysteine	C	3.1	1.5	1.7
Glutamic acid	E	3.1	3.1	3.1
Histidine	H	3.1	4.6	4.2
Isoleucine	I	3.1	3.4	3.7
Lysine	K	3.1	2.3	2.5
Methionine	M	3.1	3.1	3.1
Phenylalanine	F	3.1	2.7	3.5
Tryptophan	W	3.1	2.3	2.4
Tyrosine	Y	3.1	3.6	4.0

Simulated naïve library displays no clustering

To investigate the appearance of a truly random library, a naïve library was simulated using the same number of input sequences. Figure S1 shows PCA plot and heatmap of the simulated data set, where only one cluster is formed. The heatmap shows small differences for each amino acid at every position, when comparing the observed frequency to the expected frequency.

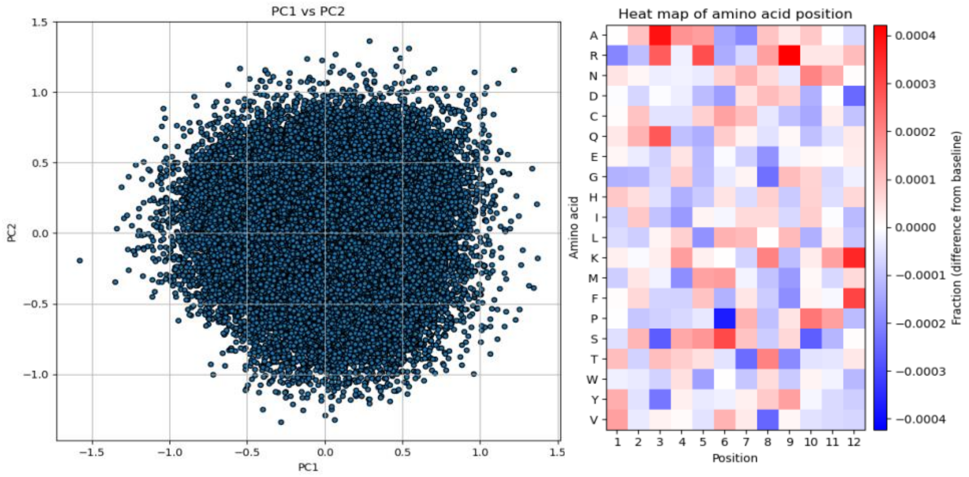

Figure S1: Simulation of a naïve peptide phage display library

A simulation of a naïve library with the same number of reads (6,940,916) was performed. Principal component analysis (PCA) was conducted and a heatmap of the expected positional frequencies were made. The amino acids are incorporated according to the NNK strategy.
